# Estimation of Newborn Risk for Child or Adolescent Obesity: Lessons from Longitudinal Birth Cohorts

**DOI:** 10.1371/journal.pone.0049919

**Published:** 2012-11-28

**Authors:** Anita Morandi, David Meyre, Stéphane Lobbens, Ken Kleinman, Marika Kaakinen, Sheryl L. Rifas-Shiman, Vincent Vatin, Stefan Gaget, Anneli Pouta, Anna-Liisa Hartikainen, Jaana Laitinen, Aimo Ruokonen, Shikta Das, Anokhi Ali Khan, Paul Elliott, Claudio Maffeis, Matthew W. Gillman, Marjo-Riitta Järvelin, Philippe Froguel

**Affiliations:** 1 Unité Mixte de Recherche 8199, Centre National de Recherche Scientifique (CNRS) and Pasteur Institute, Lille, France; 2 Regional Centre for Juvenile Diabetes, Obesity and Clinical Nutrition, University of Verona, Verona, Italy; 3 Department of Clinical Epidemiology and Biostatistics, McMaster University, Hamilton, Canada; 4 Obesity Prevention Program, Department of Population Medicine, Harvard Medical School and Harvard Pilgrim Health Care Institute, Boston, Massachusetts, United States of America; 5 Institute of Health Sciences and Biocenter, University of Oulu, Oulu, Finland; 6 Department of Children, Young People and Families, National Institute for Health and Welfare, Helsinki, Finland; 7 Institute of Clinical Medicine/Obstetrics and Gynecology, University of Oulu, Oulu, Finland; 8 Finnish Institute of Occupational Health, Helsinki, Finland; 9 Department of Clinical Sciences and Clinical Chemistry, University of Oulu, Oulu, Finland; 10 Department of Epidemiology and Biostatistics, School of Public Health, Imperial College, London, United Kingdom; 11 Centre for Environment and Health, School of Public Health, Imperial College, London, United Kingdom; 12 Department of Life Course and Services, National Institute for Health and Welfare, Oulu, Finland; 13 Department of Genomics of Common Disease, School of Public Health, Imperial College, London, United Kingdom; Scientific Directorate, Bambino Hospital, Italy

## Abstract

**Objectives:**

Prevention of obesity should start as early as possible after birth. We aimed to build clinically useful equations estimating the risk of later obesity in newborns, as a first step towards focused early prevention against the global obesity epidemic.

**Methods:**

We analyzed the lifetime Northern Finland Birth Cohort 1986 (NFBC1986) (N = 4,032) to draw predictive equations for childhood and adolescent obesity from traditional risk factors (parental BMI, birth weight, maternal gestational weight gain, behaviour and social indicators), and a genetic score built from 39 BMI/obesity-associated polymorphisms. We performed validation analyses in a retrospective cohort of 1,503 Italian children and in a prospective cohort of 1,032 U.S. children.

**Results:**

In the NFBC1986, the cumulative accuracy of traditional risk factors predicting childhood obesity, adolescent obesity, and childhood obesity persistent into adolescence was good: AUROC = 0·78[0·74–0.82], 0·75[0·71–0·79] and 0·85[0·80–0·90] respectively (all p<0·001). Adding the genetic score produced discrimination improvements ≤1%. The NFBC1986 equation for childhood obesity remained acceptably accurate when applied to the Italian and the U.S. cohort (AUROC = 0·70[0·63–0·77] and 0·73[0·67–0·80] respectively) and the two additional equations for childhood obesity newly drawn from the Italian and the U.S. datasets showed good accuracy in respective cohorts (AUROC = 0·74[0·69–0·79] and 0·79[0·73–0·84]) (all p<0·001). The three equations for childhood obesity were converted into simple Excel risk calculators for potential clinical use.

**Conclusion:**

This study provides the first example of handy tools for predicting childhood obesity in newborns by means of easily recorded information, while it shows that currently known genetic variants have very little usefulness for such prediction.

## Introduction

Childhood and adolescent overweight and obesity, which are leading causes of early type 2 diabetes and cardiovascular disease, have become major public health problems both in westernized and more recently in developing countries [1]. Traditional approaches for the management of overweight and obesity have had poor long term efficacy and therefore prevention is currently the most promising strategy for controlling the obesity epidemic [1].

Prevention of obesity should start as early as possible after birth. Longitudinal studies have shown a strong association between early infancy weight gain rate or adiposity and childhood and even adult body weight, fat mass and body mass index (BMI) [2]–[3]. Moreover, the efficacy of preventive behavioural and nutrition interventions targeting school children, either in primary schools or at home, is very limited [4]–[5]. Finally, in many countries pre-school and school children are already burdened by a high prevalence of overweight or obesity [4].

Assessing the risk for future overweight or obesity in newborns may be a basis for focused preventive interventions for at-risk individuals during the very first months of their life. Even though several sociodemographic and anthropometric predictors, as well as several common genetic variants, have been associated with childhood overweight/obesity, no longitudinal study has attempted to explore the cumulative predictive properties of these known early life risk factors, or to propose possible tools to predict childhood obesity at birth [6]–[20].

We aimed to build such predictive algorithms for the early identification of newborns at an increased risk for childhood and adolescent overweight/obesity. For this purpose, we estimated the ability of clinical, socio-demographic, and genetic risk factors to predict childhood and adolescent overweight/obesity in a large Finnish birth cohort. We then confirmed the promising usefulness of socio-demographic and anthropometric factors in predicting childhood obesity in two independent paediatric cohorts.

## Methods

### Ethics Statement

The study conducted on the NFBC1986 cohort was approved by the Ethical Committee of Northern Ostrobothnia Hospital District. The retrospective study of the Veneto cohort was approved by the Ethical Committee of the University of Verona and Project Viva was approved by the Human subjects Committees of Harvard Pilgrim Health Care, Brigham and Women’s Hospital, and Beth Israel Deaconess Medical Center.

Written informed consent was obtained from parents or guardians of all participants and all clinical investigations were conducted according to the principles expressed in the Declaration of Helsinki.

### Subjects

#### Development sample

The Northern Finland Birth Cohort 1986 (NFBC1986) (http://kelo.oulu.fi/NFBC) was followed prospectively from 12^th^ gestational week and several well known early risk factors for childhood obesity were recorded systematically. Participants who had their weight and height recorded at seven and sixteen years of age and met data completeness criteria (see below, N = 4,032) were used to build the models. We separately predicted childhood obesity (obesity at 7 years of age), childhood overweight/obesity (overweight or obesity at 7 years of age), adolescent obesity (obesity at 16 years of age), adolescent overweight/obesity (overweight or obesity at 16 years of age), and the severe sub-phenotypes of childhood obesity persistent into adolescence (obesity at 7 and 16 years of age) and childhood overweight/obesity persistent into adolescence (overweight or obesity at 7 and 16 years of age) (Table 1, Table S1–S2). Overweight and obesity were defined by the IOTF BMI cut-offs [21].

The traditional predictors used for building the predictive models (gender, pre-pregnancy parental BMI, parental professional category, single parenthood, gestational weight gain, pre-pregnancy maternal smoking, gestational smoking, number of household members, birth weight) were *a-priori* selected among all available baseline NFBC1986 variables according to their association with early obesity in previous literature (Table 1) [2], [6]–[11]. Forty-four obesity predisposing single-nucleotide polymorphisms (SNPs) were selected according to the following criterion: genome-wide significant level of association (P<5×10^−8^) for BMI and/or obesity reported in a population of European ancestry [12]–[20]. Genotyping was performed by TaqMan (Applied Biosystems, Foster City, CA): the average genotyping success was of 99.4% (95.1–100) and the average consensus rate from 255 duplicates was 99.8% (99.2–100) (Table S3, S4, S5, S6).

Five SNPs were discarded during the genotyping procedure, since they did not pass the genotyping quality control criteria, leaving 39 SNPs. All 39 SNPs were in Hardy-Weinberg equilibrium (P>0.05). We assumed an additive model and constructed a cumulative genotype score by summing the number of risk alleles (0–78).

#### Validation samples

We used a school-based retrospective sample of 1,503 children aged 4–12 from Veneto, Italy, as one of the two validation samples to explore whether results from the NFBC1986 could be applied to a European paediatric cohort contemporary to the NFBC1986, with similar obesity prevalence (4%) but different cultural background [22]. The second validation set used was a prospective sample of 1032 children (7 years) from Massachusetts (United States) from the Project Viva (http://www.dacp.org/viva/index.html) to explore whether results would remain valid when applied to a very recent U.S. child cohort, with higher obesity prevalence (8%) and very different cultural background. Genetic variants were not available for the validation analyses. All children meeting the international criterion for obesity definition at the time of recruitment in the Italian sample and at 7 years of age in the U.S. sample were classified as affected by childhood obesity [21].

**Table 1:** Characteristics of the NFBC1986 cohort.

BASELINE	
**Males**	1,917 (47.5)
**Mother’s age (years)**	28.5 (16.9–50.8)
**Father’s age (years)**	30.8(17.9–59.8)
**Single parenthood**	113 (2.9)
**Mothers smoking before pregnancy**	994 (24.7)
**Mothers smoking during pregnancy**	737 (18.3)
**Maternal BMI before pregnancy**	22.3 (13.2–48.2)
**Paternal BMI**	24.0 (16.9–41.3)
**Maternal professional category**	
4 Professional/entrepreneur	277 (6.9)
3 Skilled-non manual	866 (21.5)
2 Skilled-manual	1,625 (40.3)
1 Unskilled/apprentice/unemployed	1,264 (31.3)
**Paternal professional category**	
4 Professional/entrepreneur	545 (13.5)
3 Skilled-non manual	856 (21.2)
2 Skilled-manual	1,934 (48)
1 Unskilled/apprentice/unemployed	691 (17.1)
**Household members**	3.6 (1–18)
**Maternal percentage weight gain during pregnancy**	23.3 (−12.0–111.6)
**Gestational age**	39.3 (27–43)
**Birth weight (kg)**	3.560 (0.740–5.560)
**Genetic score**	37.2 (25–50)
**OUTCOME**	
**Childhood obesity (at 7 years of age)**	121 (3)
**Childhood overweight/obesity (at 7 years of age)**	645 (16)
**Adolescent obesity (at 16 years of age)**	163 (4)
**Adolescent overweight/obesity (at 16 years of age)**	678 (17)
**Persistent childhood obesity (at both 7 and 16 years of age)**	47(1)
**Persistent childhood overweight/obesity (at both 7 and 16 years of age)**	331 (8)

Data are given as MEAN (range) or as N (percentage).

### Statistical Analysis

#### Development phase

Predictive models were fitted by stepwise logistic regression analysis (criterion for variable entry: p<0.05, for variable removal: p>0.10) using traditional risk factors only, genetic score only and traditional risk factors plus genetic score for each obesity outcome. Each risk factor entering the analysis as continuous or ordinal scale variable showed a linear relationship with the logit-risk of childhood obesity in a preliminary linear regression analysis. For persistent childhood obesity, not all the *a priori* selected traditional predictors were used for the stepwise analysis but only the five with the strongest association with persistent childhood obesity in a preliminary univariate analysis, in order to avoid possible model over-fitting due to the relatively small number (forty-seven) of outcome events.

The discrimination accuracy of each model was evaluated by the area under the receiver operating curve (AUROC) of the modeled risk [23]. Models with AUROCs larger than 0.7 were considered potentially clinically useful and those with AUROCs larger than 0.8 were considered to have excellent accuracy [23]. The model calibration, that is the “precision” or correlation between the predicted and observed event rate, was assessed by the Hosmer-Lemeshow test [23]. The possible accuracy improvement associated with adding the genetic score to the traditional risk factors was evaluated by calculating the integrated discrimination improvement (IDI) compared to the traditional risk factors alone [24].

For each model a risk threshold was arbitrarily adopted at the 75^th^ percentile of the modeled risk, identifying the top 25% as being at increased risk and the thresholds’ predictive properties (sensitivity, specificity and predictive values) were calculated.

An average of 1.67% (0–11.4%) of data was missing for each traditional risk factor, while an average of 0.72% (0–4.95%) of genotypes was missing for each SNP. We included participants with zero or one missing traditional baseline variable and three or fewer missing SNPs. Multiple imputation was performed for the remaining missing values, in order to avoid possible bias associated with missing potentially important information [25]. Win MICE (Multiple Imputation by Chain Equations) V0.1. was used for multiple imputation [25]. By the MICE procedure, imputed values for missing data are drawn from modelling them on the basis of the other considered variables, with logistic regression if the variable to impute is dichotomous, polytomous logistic regression if it is categorical with three or more categories and with linear regression if it is continuous [25]. So each missing value is replaced by an estimated value modelled on the other variables. Indeed, the method estimates a distribution of each missing variable, taking all aspects of uncertainty in the imputations into account. From this distribution, values are sampled and filled in for the missing data. So every imputation cycle produces, for each missing data, one estimated value sampled among several possible ones, giving rise to a unique dataset which can not be reproduced by following imputation cycles [25].

Five imputation cycles were run so that five values were imputed for each missing datum to get variation in the imputed values, thus reflecting the uncertainty introduced by imputation itself. Inference was based on the five resulting datasets [25]: areas under AUROCs were obtained by averaging the five single data sets coefficients, while 95% confidence intervals were delimited by the two overall most extreme boundaries, the lowest and the highest [25]. All the coefficients, the AUROCs and the 95% C.I. boundaries were identical up to the first or second decimal for any considered variable across the five datasets.

#### Validation and replication phase

Only the model developed for childhood obesity was used for validation because the model for prediction of childhood overweight/obesity was not considered accurate enough to be clinically useful and the models concerning adolescent phenotypes required older cohorts than Veneto and Project Viva. The NFBC1986 equation was applied to the validation cohorts after recalculation of the intercept according to the cohort-specific phenotype prevalence and mean values of predictors. In the Veneto sample, number of household members and gestational smoking were not available.

A replication analysis was also performed in which the model for childhood obesity was re-built in the two validation samples by stepwise logistic regression using the available traditional risk factors.

Statistics were performed with R 2.11.0 (www.r-project.org), SPSS.18 (IBM Company, Chicago, Illinois) and SAS 9.3 (SAS Institute, Cary, North Carolina).

## Results

Parental BMI, birth weight, maternal gestational weight gain, number of household members, maternal professional category and smoking habits were independent predictors of all or most of the six obesity outcomes (Table 2–3).

**Table 2:** Stepwise multiple logistic models for prediction of overweight phenotypes: ORs and p values associated with predictors, AUROC and P of Hosmer-Lemeshow test in the final models (bold characters) and AUROCs and P of Hosmer-Lemeshow of each step (italic characters).

	OR in the finalcumulative model	P	AUROC whenterm is added	P of H-L test whenterm is added
***Childhood Overweight-Obesity***				
Maternal BMI	**1.13 (1.10–1.16)**	**<0.001**	*0.63 (0.60–0.65)*	*<0.001*
Paternal BMI	**1.11 (1.08–1.15)**	**<0.001**	*0.65 (0.62–0.67)*	*0.042*
N of household members	**0.88 (0.84–0.93)**	**<0.001**	*0.66 (0.64–0.68)*	*0.023*
Gestational weight gain	**1.02 (1.01–1.03)**	**<0.001**	*0.66 (0.64–0.69)*	*0.015*
Birth weight	**1.45 (1.22–1.73)**	**<0.001**	*0.67 (0.65–0.69)*	*0.29*
Maternal smoking	**1.28 (1.05–1.57)**	**0.013**	**0.67 (0.65–0.69)**	**0.46**
***Adolescent Overweight-Obesit*** **y**				
Maternal BMI	**1.17 (1.14–1.20)**	**<0.001**	*0.66 (0.63–0.67)*	*0.05*
Paternal BMI	**1.12 (1.09–1.15)**	**<0.001**	*0.68 (0.66–0.70)*	*0.13*
Gestational weight gain	**1.02 (1.00–1.03)**	**0.001**	*0.70 (0.68–0.72)*	*<0.001*
N of household members	**0.90 (0.86–0.95)**	**<0.001**	*0.70 (0.68–0.72)*	*<0.001*
Birth weight	**1.31 (1.12–1.53)**	**<0.001**	*0.71 (0.69–0.72)*	*0.07*
Maternal occupation	**0.75 (0.60–0.93)**	**0.009**	*0.71 (0.69–0.73)*	*0.20*
Maternal smoking	**1.28 (1.06–1.54)**	**0.009**	**0.71 (0.69–0.73)**	**0.09**
***Persistent Childhood Overweight-Obesity***				
Maternal BMI	**1.18 (1.14–1.22)**	**<0.001**	*0.69 (0.66–0.72)*	*0.001*
Paternal BMI	**1.14 (1.10–1.19)**	**<0.001**	*0.72 (0.69–0.75)*	*0.002*
Gestational weight gain	**1.03 (1.02–1.04)**	**<0.001**	*0.73 (0.70–0.75)*	*0.009*
N of household members	**0.88 (0.82–0.95)**	**<0.001**	*0.73 (0.71–0.75)*	*0.001*
Maternal occupation	**0.57 (0.42–0.77)**	**<0.001**	*0.74 (0.72–0.77)*	*0.01*
Birth weight	**1.41 (1.12–1.77)**	**0.003**	*0.74 (0.72–0.77)*	*0.06*
Gestational smoking	**1.45 (1.09–1.94)**	**0.011**	**0.75 (0.73–0.78)**	**0.07**

The equations to estimate the risk for the obesity outcomes from these traditional risk factors are represented in supporting information (Dataset S1).

**Table 3:** Stepwise multiple logistic models for prediction of obesity phenotypes: ORs and p values associated with predictors, AUROC and P of Hosmer-Lemeshow test in the final models (bold characters) and AUROCs and P of Hosmer-Lemeshow of each step (italic characters).

	OR in the finalcumulative model	P	AUROC when termis added	P of H-L test whenterm is added
***Childhood Obesity***				
Paternal BMI	**1.19 (1.13–1.27)**	**<0.001**	*0.68 (0.64–0.73)*	*0.39*
Maternal BMI	**1.13 (1.08–1.17)**	**<0.001**	*0.74 (0.70–0.78)*	*0.06*
N of household members	**0.73 (0.63–0.84)**	**<0.001**	*0.77 (0.73–0.80)*	*0.007*
Birth weight (kg)	**2.12 (1.48–3.04)**	**<0.001**	*0.77 (0.73–0.80)*	*0.47*
Maternal occupation	**0.50 (0.31–0.79)**	**0.003**	*0.77 (0.73–0.81)*	*0.57*
Gestational smoking	**1.84 (1.20–2.81)**	**0.005**	**0.78 (0.74–0.82)**	**0.52**
***Adolescent Obesity***				
Maternal BMI	**1.18 (1.13–1.23)**	**<0.001**	*0.67 (0.63–0.71)*	*0.13*
Paternal BMI	**1.16 (1.10–1.22)**	**<0.001**	*0.70 (0.66–0.74)*	*0.29*
N of household members	**0.83 (0.74–0.92)**	**0.001**	*0.73 (0.69–0.76)*	*0.29*
Maternal occupation	**0.47 (0.32–0.69)**	**<0.001**	*0.74 (0.71–0.78)*	*0.81*
Gestational weight gain (%)	**1.03 (1.01–1.05)**	**0.001**	**0.75 (0.71–0.79)**	**0.69**
***Persistent Childhood Obesity***				
Paternal BMI	**1.23 (1.13–1.34)**	**<0.001**	*0.69 (0.61–0.76)*	*0.93*
Maternal BMI	**1.14 (1.07–1.21)**	**<0.001**	*0.81 (0.76–0.87)*	*0.32*
Birth weight	**2.30 (1.29–4.08)**	**0.005**	*0.82 (0.76–0.88)*	*0.06*
Maternal occupation	**0.31 (0.16–0.57)**	**<0.001**	*0.84 (0.79–0.89)*	*0.55*
Single parenthood	**4.27 (1.39–13.12)**	**0.011**	**0.85 (0.80–0.90)**	**0.33**

**Figure 1:**
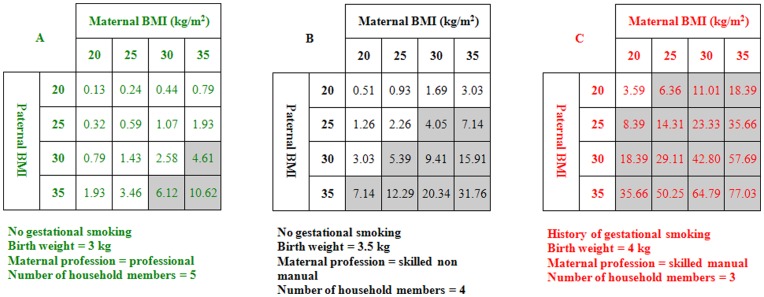
Estimates of risk percentages for childhood obesity for given pairs of parental BMIs according to the NFBC1986 equation. Estimates are provided for three different combinations of birth weight, maternal professional category, number of household members and maternal gestational smoking, corresponding to three progressively higher risk backgrounds. Grey cells correspond to risk estimates within the highest risk quartile in the overall population.

Discrimination accuracy of the risk calculation from traditional risk factors was excellent for persistent childhood obesity (AUROC = 0.85[0.80–0.90], p<0.001), clinically meaningful for persistent childhood overweight/obesity (AUROC = 0.75[0.73–0.78], p<0.001), childhood obesity (AUROC = 0.78 [0.74–0.82], p<0.001), adolescent obesity (AUROC = 0.75[0.71–0.79], p<0.001) and adolescent overweight/obesity (AUROC = 0.71[0.69–0.73], p<0.001), and below the threshold for clinical usefulness for childhood overweight/obesity (AUROC = 0.67[0.65–0.69], p<0.001) (Figure 1 and Table 2–3) (23). All of the six models developed from traditional risk factors were adequately calibrated (all p for Hosmer-Lemeshow test >0.05).

Parental BMI was the main contributor to discrimination accuracy while other predictors contributed moderately to the model discrimination effectiveness but increased the overall model calibration (Table 2–3).

For any given pair of parental BMIs, estimation of the probability of childhood obesity varied greatly, depending on the combination of other predictors (Figure 1).

Genetic score was an independent predictor of all of the six considered outcomes, with ORs associated with unitary score increase ranging from 1.05[1.03–1.08] to 1.09[1.03–1.14] (0.05> all P>4 × 10^−8^) but its discrimination accuracy was poor, with AUROCs ranging from 0.56[0.54–0.58] to 0.59[0.54–0.64] (Table S7). Adding the genetic score to the traditional risk factors did not produce better AUROCs than using traditional risk factors alone and was associated with modest IDIs not larger than 1% (Figure S1). The genetic score composed of only the twenty SNPs identified for childhood obesity traits exhibited similar associations with early obesity phenotypes (Table S8). Then only the models developed from traditional risk factors were taken into consideration for further analyses. Predictive properties of the risk thresholds corresponding to the highest risk quartile for each obesity phenotype are represented in Table 4. Positive predictive values were low, due to the low prevalence of predicted conditions, while negative predictive values were high (Table 4).

**Table 4:** Risk threshold and predictive properties corresponding to the 75° percentile of calculated risk for the obesity phenotypes in the NFBC1986.

	Riskthreshold	Sensitivity %	Specificity %	Positive Predictivevalue %	Negative Predictivevalue %
**Childhood Obesity**	0.036	72 [65–79]	76.5 [75–78]	9 [7]–[11]	99 [98.5–99.5]
**Adolescent Obesity**	0.048	66 [59–73]	77 [75.5–78.5]	11 [9]–[13]	98 [97–99]
**Persistent childhood obesity**	0.011	79 [69–89]	75.5 [74–77]	4 [3]–[5]	99.5 [98–100]
**Childhood overweight/obesity**	0.194	45 [37–53]	79 [77–81]	29 [26]–[32]	88 [87–89]
**Adolescent overweight/obesity**	0.210	49 [45–53]	80 [78.5–81.5]	33 [30]–[36]	88.5 [87–90]
**Persistent childhood overweight/** **obesity**	0.097	63 [58–68]	78 [76.5–79.5]	21 [18]–[24]	96 [95–97]

The version of the NFBC1986 equation for childhood obesity lacking gestational smoking and number of household members (AUROC = 0.73[0.69–0.77] in the NFBC1986) had an AUROC = 0.70[0.63–0.77] (p<0.001) when applied to the Veneto cohort, with acceptable calibration accuracy (p for Hosmer-Lemeshow test = 0.12).

The NFBC1986 equation for childhood obesity had an acceptable AUROC = 0.73[0.67–0.80] (p<0.001) when applied to the project Viva children. However, calibration in the Project Viva sample was not satisfactory (p for Hosmer-Lemeshow test = 0.02).

The VENETO equation, i.e. the equation to predict childhood obesity issued from the Italian sample (model replication), included parental BMIs and gender (Dataset S1), had an AUROC of 0.74[0.69–0.79] (p<0.001) in the Veneto sample and was adequately calibrated (p for Hosmer-Lemeshow test = 0.11).

The Project Viva equation, i.e., the equation to predict childhood obesity issued from the U.S. sample (model replication), included parental BMI, race, gestational smoking and gestational weight gain (Dataset S1), had an AUROC of 0.79[0.73–0.84] (p<0.001) in the Project Viva sample and was adequately calibrated (p for Hosmer-Lemeshow test = 0.91).

The three equations predicting childhood obesity in the three studied cohorts were converted in an electronic automatic risk calculator for potential clinical use (Dataset S2).

## Discussion

Our study provides the first example of predictive tool for assessing the risk of developing early obesity phenotypes, based on readily available traditional risk factors about newborns. The potential inclusion of genetic variants was explored, but due to their modest contribution to predictive accuracy, they were not included in the final models.

Analysis of the NFBC1986 showed that traditional risk factors performed better in prediction of severe rather than mild obesity phenotypes. Importantly, the predictive accuracy of the models did not decline from childhood to adolescence, suggesting that the association between the traditional risk factors and obesity is stable until early adulthood. This is consistent with recent evidence about the relationship between single early risk factors and adolescent and adult obesity [6], [9], [10]. The risk of childhood obesity was largely driven by parental BMI. However, other predictors moderately improved the discrimination accuracy and increased the exactitude of risk estimation. They also produced large ranges of possible risk estimates for any given parental BMI, significantly improving risk classification at any level of parental BMI (Table 2–3, Figure 1 and Dataset S2).

Predictive tools need to satisfy important requisites before they can be applied in clinical settings. First, significant preventive advantages should derive from prediction. Although medical societies have been called on to provide reasonable guidance on prevention based on available data and the American Academy of Paediatrics has recently underlined the emergent need of finding effective clinical tools to enable primary care providers to contribute to obesity prevention [26]–[27], there is no compelling evidence of any efficient obesity preventive strategy involving infancy. Then, robust trials proving the effectiveness of strategies of early prevention are still needed to justify the adoption of early obesity prediction in the everyday clinical practice. Should trials prove the efficacy of preventive strategies implying special interventions going beyond paediatric counselling and public health campaigns routinely provided to the general population, a predictive tool like that proposed here would offer the important advantage to exclude a large proportion of infants from such interventions, thanks to its good negative predictive value. This would improve the cost/effectiveness ratio of preventive actions.

However few available controlled prevention trials suggest that interventions directly involving parents of pre-school children outside education settings are more effective than school or community-based interventions targeting later ages, supporting the hypothesis that involving parents in the prevention of their offspring’s obesity as early as possible is likely to be a good strategy [1]. In this view, it has been suggested that « Let’s Move » against child obesity campaign, which is a U.S. government-sponsored obesity prevention program targeting children aged 2–10, might be more effective if children under 2 could be identified as prevention targets [4].

Parents of newborns are particularly sensitive to information given about their child’s health. Once informed of their baby’s increased risk for obesity, they might be more receptive to routine advice provided from birth during the first two years of life within population-wide prevention: breastfeeding, feeding on demand, weaning no earlier than the sixth month with recommended meal patterns and food portions, avoiding of television and sugar-sweetened beverages [28]. Moreover, families of newborns at risk could be enrolled in more intensive schedules of growth monitoring and nutritional counselling than those offered to general population, in order to avoid excessive weight gain in infancy. Encouraging strategies aiming at significantly decreasing energy intake in infants should be avoided however, both because of the well known difficulties encountered by parents in doing it and because of potential, unknown harmful effects of an early caloric restriction. In contrast, recent evidence suggests that some preventive strategies prevention of obesity based on educating mothers could be useful to limit excessive infant weight gain promoting appropriate maternal responses to satiety cues and decreasing non-responsive feeding behaviours which over-ride satiety cues, such as food rewards, non food rewards to encourage infant to eat, etc… [29]. Such strategies do not imply a direct food restriction, but rather a limitation of “passive” (not hunger-driven) infant over-eating.

Obviously, even in case of proved efficacy of early obesity prevention, the targeted approach should also be carefully assessed by means of trials with a “focused intervention” design, before any dissemination of the early obesity prediction into broad clinical practice. In fact, targeted approach might also imply deleterious effects, among which, for example, stigmatization of families of infants classified as “at risk” or false reassurance of other families. Indeed, early prediction should not mean a “diagnostic” attitude towards any of the two categories of families. In particular, the assessment of age and BMI at adiposity rebound, which are good predictors of childhood and adult obesity, should be carried on in young children in order to optimize the overall detection rate of those likely to become obese and possibly sensitize families previously “missed” by the neonatal score [30].

Accuracy is another important requisite for a predictive tool. The model predicting persistent obesity had excellent accuracy (AUROC = 0.85) while the models predicting obesity and persistent overweight had clinically useful discrimination accuracy (AUROCs = 0.75 to 0.78) [23], similar to that of widely used tools for predicting multifactor medical conditions, such as the Framingham risk score for coronary heart disease (AUROC = 0.74 to 0.77 depending on gender and type of scoring adopted) [31]. Due to low prevalence of the obesity phenotypes in the NFCB1986, the fourth quartiles of predicted risk had low to moderate prevalence of cases even if they “captured” most or a high percentage of cases (low positive predictive value despite good sensitivity) (Table 4). This represents a possible drawback of preventive strategies based on risk assessment [32]. Nevertheless, risk thresholds conceived for prediction and focused prevention are not required to be “diagnostic” but rather cost-effective. Thus, the criteria we propose to select newborns at risk for obesity, could have a strong impact on public health, despite their low specificity/positive predictive value, because they could justify cost-effective preventive strategies on a subsection of the general population, similarly to several sensitive though little specific selective criteria used for widespread preventive interventions, such as: age higher than 30 years as criterion to recommend pap test against cervical cancer, age higher than 50 years as criterion to recommend the faecal occult blood test against colon cancer, etc…[33]–[34]. The adequate discrimination and calibration accuracy achieved by the equations presented in the manuscript imply that a high percentage of future obese children (more than two-thirds), is included in the highest quartile of calculated risk. Thus, using the highest risk quartile of calculated risk as selective criterion would allow focused preventive strategies to reach 70–75% of potential future cases though involving only 25% of newborns. Should these strategies have just about 50% effectiveness, the number of future obese children would have a 35–38% decrease, which would represent much greater success compared with results obtained to date by large scale preventive strategies involving later infancy and childhood [5]

The models using traditional risk factors had good calibration, which suggests that it may be possible to use the newborns’ calculated risks in addition to the two risk categories. This would add precision to prediction and potential further effectiveness to related prevention

Finally, the equations we present use easily accessible information, do not incur additional costs to clinical care, and only require minimal time to calculate, if converted into simple automatic calculators like those we propose in the Supporting Information. Such electronic risk calculators could be part of an electronic medical record system and/or be housed within computer-assisted standardised programs of obesity prevention, which are promising tools for the prevention and care of paediatric obesity [35].

The results of the validation/replication analyses allow for important considerations. First of all, traditional risk factors have a good cumulative accuracy (AUROC = 0.79) in the recent U.S. paediatric cohort, which has a significantly higher prevalence of childhood obesity than the NFBC1986. This demonstrates that the environmental pressure towards obesity has not weakened the role of early risk factors. Moreover, it supports the hypothesis that, at the current phase of the obesity pandemic, the use of “familial and personal” risk factors for early prediction may be useful, in addition to population wide interventions, in those regions, like Massachusetts, where the prevalence of obesity is still moderate and characterised by ethnic and social disparities rather than influenced by country-related risk factors [36]. In these regions, focused preventive strategies based on personal risk stratification may effectively integrate large scale interventions based on nation wide characteristics [32], [36]. Interestingly, since 2010 the U.S Government has been supporting a preventive strategy against childhood obesity involving low-income children from Boston (http://www.cdc.gov/CommunitiesPuttingPreventiontoWork/communities/profiles/both-ma_boston.htm), indicating efforts towards focused prevention. Employing focused strategies involving newborns whose risk is high according to diverse factors beyond social parameters, could lead to earlier, more effective prevention of overweight/obesity in children.

The NFBC1986 equation for childhood obesity proved to keep acceptably discriminative when applied to both the validation cohorts, but showed a lost of calibration when applied to the Viva cohort, suggesting that its adoption in the U.S. would have acceptable validity to discriminate newborns at risk for early obesity but not to perform exact risk estimations. This is probably due to inconsistency of some predictors, such as maternal professional category and number of household members. Accordingly, the Project Viva equation lacks these variables while it includes race, which is not present among obesity predictors in the NFBC1986 equation, because of the high ethnical homogeneity of the NFBC1986. Inconsistency of the role of SES variables across different populations is expected and it is the main reason why it would be very difficult to build a highly accurate and calibrated score that also has complete widespread validity [36].

Overall, the validation analysis suggests that “local” equations, including parental BMI but also other locally important early predictors, may have good accuracy in predicting childhood obesity at birth, even in countries like the U.S., with high environmental pressure towards early weight excess, and should be preferred, whenever possible, to the universal adoption of the NFBC1986 equation. Interestingly, parental BMI, which partly reflects the degree of familial genetic predisposition to obesity, had very similar effect size and accuracy in the three studied cohorts, consistently with the evidence that the growing obesity epidemic has not lowered the heritability of childhood adiposity [37].

Our study also explored, with the largest list of obesity-SNPs ever used, the performance of genetics in predicting early obesity phenotypes, showing very modest predictive accuracy of the assessed genetic variants, consistently with previous evidence on adult obesity [20]. Even if a modest predictive accuracy of the studied genetic variants was expected, the accuracy estimates obtained in this study rule out, for the first time, the hypothesis that genetics may perform a little better in predicting early obesity than adult obesity, due to presumed lower impact of environmental determinants during childhood than later in life. This result is consistent with recent evidence that polygenic risk and BMI show substantially similar correlation coefficients between childhood and adulthood and further contributes to the growing evidence that common genetic variants are not yet “ready for use” for the prediction of several complex diseases, due to the still small proportion of heritability explained by the newly discovered variants [30], [38]. It is possible that next-generation sequencing techniques will reduce significantly the gap of “missing heritability” of obesity, identifying rare causative variants and clarifying the role of epigenetics by the genome-wide characterisation of DNA methylation patterns in foetuses or infants developing later obesity or not [39].

Finally, the most important evidence obtained by including currently known SNPs in our analyses is that not only common genetic variants have very low accuracy in predicting early obesity but also they produce a very little improvement of the prediction when combined with clinical factors. This is particularly important because although the notion that genetic variants have poor value in predicting common diseases is quite well established, the possible utility of including polygenic risk scoring within management strategies for complex diseases is a topical subject of current research and genetic testing services including obesity are being offered to consumers by private companies [39]–[40].

The main limitations of our manuscript are the lack of external validation for the equations predicting adolescent and persistent obesity, due to the young age of our validation cohorts and the use, in one of the validation analyses, of a retrospective paediatric cohort with some variables lacking and an age of assessment not perfectly corresponding to that of the original cohort (4–12 years versus 7 years).

The main strengths include: the novelty and the potential strong public health impact of multivariate obesity predicting tools valid for newborns; the optimization of results reliability and robustness by the adoption of several recommended methods shown recently to be lacking in several recent high impact prediction studies [41]: external geographical and temporal validation (for the model predicting childhood obesity), use of multiple imputation for missing values, avoidance of predictor dichotomisation, assessment of models calibration accuracy, avoidance of model over-fitting.

In summary, our study provides the first example of at birth prediction of early obesity by means of traditional, routinely available risk factors and should guide future efforts towards randomized trials of very early preventive approaches for identified high risk individuals to help combat the obesity epidemic.

## Supporting Information

Figure S1 **ROC curves of combined traditional risk factors (blue), genetic score (beige) and traditional risk factors + genetic score (green) predicting six obesity outcomes in the NFBC1986.** Integrated discrimination improvements (IDIs) associated with adding the genetic score to the traditional risk factors are also provided.
(TIF)

Table S1 **Metabolic differences between obese adolescents with or without a history of childhood obesity in the NFBC1986.**
(DOC)

Table S2 **Metabolic differences between overweight/obese adolescents with or without a history of childhood overweight/obesity in the NFBC1986.**
(DOC)

Table S3 **SNPs selected for building the genotype score with the relative genotyping quality control parameters.**
(DOC)

Table S4 **Associations between single SNPs and childhood obesity and overweight/obesity in the NFBC1986.**
(DOC)

Table S5 **Associations between single SNPs and adolescent obesity and overweight/obesity in the NFBC1986.**
(DOC)

Table S6 **Associations between single SNPs and persistent obesity and overweight/obesity in the NFBC1986.**
(DOC)

Table S7 **Association, discrimination and calibration parameters of the 39-SNPs genetic score predicting the six obesity outcomes in the NFBC1986.**
(DOC)

Table S8 **Association, discrimination and calibration parameters of the genetic score composed of the 20 “childhood obesity SNPs”^a^ predicting the six obesity outcomes in the NFBC1986.**
(DOC)

Dataset S1 **Equations predicting the obesity phenotypes from traditional risk factors.**
(DOC)

Dataset S2 **Example of automatic calculator of risk for childhood obesity.**
(XLS)
