# Supplementary figures and images for: Estimation of Newborn Risk for Child or Adolescent Obesity: Lessons from Longitudinal Birth Cohorts
Source: PLoS One. 2012 Nov 28;7(11):e49919. doi: 10.1371/journal.pone.0049919 (PMC3509134; doi:10.1371/journal.pone.0049919)

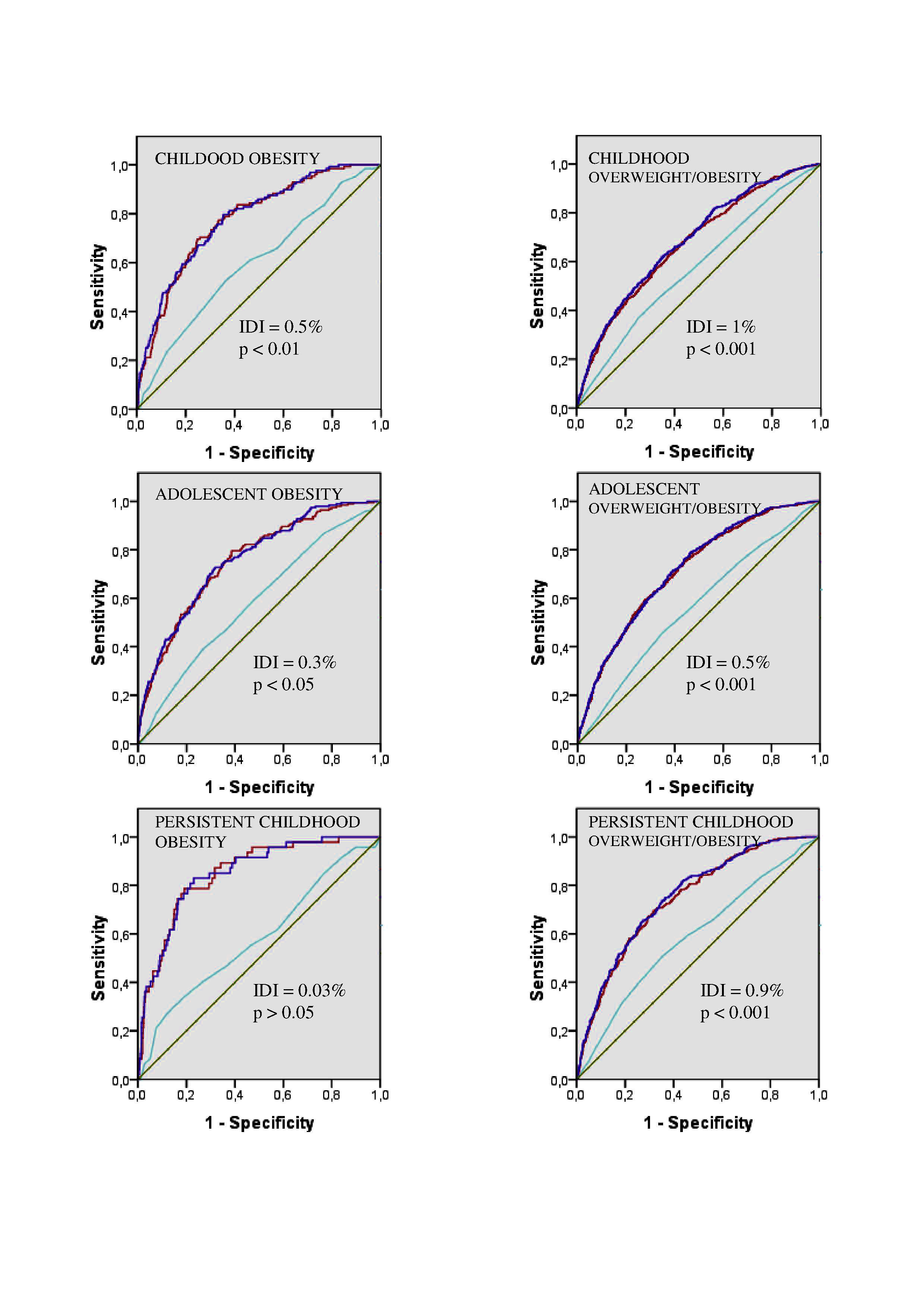

Supplement: Figure S1 — ROC curves of combined traditional risk factors (blue), genetic score (beige) and traditional risk factors + genetic score (green) predicting six obesity outcomes in the NFBC1986. Integrated discrimination improvements (IDIs) associated with adding the genetic score to the traditional risk factors are also provided. (TIF) [file pone.0049919.s001.tif]
