# Supplementary material for: Estimation of Newborn Risk for Child or Adolescent Obesity: Lessons from Longitudinal Birth Cohorts
Source: PLoS One. 2012 Nov 28;7(11):e49919. doi: 10.1371/journal.pone.0049919 (PMC3509134; doi:10.1371/journal.pone.0049919)
Supplement: Table S1 — Metabolic differences between obese adolescents with or without a history of childhood obesity in the NFBC1986. (DOC) [file pone.0049919.s002.doc]

|  | **Child obesity +** | **Child obesity -** | P |
| --- | --- | --- | --- |
| Number | 47 | 116 |  |
| BMI | 34.00(4.54) | 31.75(2.73) | 0.001 |
| Waist circumference | 103.55(10.41) | 96.48(9.98) | < 0.001 |
| Fasting plasma glucose (mmol/l) | 5.31(0.76) | 5.27(0.32) | 0.86 |
| Fasting serum insulin (UI/l) | 21.94(18.02) | 18.73(12.46) | 0.09 |
| Triglycerides (mg/dl) | 121.96(62.93) | 111.74(64.43) | 0.22 |
| HDL-cholesterol (mg/dl) | 43.92(7.68) | 47.15(8.20) | 0.03 |
| Systolic blood pressure | 127.44(14.37) | 128.11(13.81) | 0.74 |
| Diastolic blood pressure | 73.96(8.63) | 75.24(8.66) | 0.45 |
| Metabolic Syndrome^a^ carriers [Number (percentage)] | 16(34) | 18(15) | 0.02 |

“child obesity +”= obese adolescents with a history of childhood obesity (obesity at 7 years of age); “child obesity-”= obese adolescents without a history of childhood obesity (obesity at 7 years of age).

**^a^** *Metabolic syndrome was diagnosed if at least two other metabolic risk factors among impaired fasting glucose (fasting plasma glucose ≥ 100 mg/dl), high triglycerides (> 143 mg for boys and > 126 mg/dl for girls), low HDL-cholesterol (< 30 mg/dl for boys and < 35 mg/dl for girls) and systolic and/or diastolic hypertension (≥ 130 and 85 mmHg respectively) were present besides obesity. Triglycerides and HDL-cholesterol cut-offs corresponded to the 95° percentiles proposed by the AAP according to:* Tamir I, Heiss G, Glueck CJ et Al. Lipid and lipoprotein distributions in white children aged 6-19 years: the Lipid Research Clinics Program Prevalence Study. J Chronic Dis, 1981; 34: 27-39.
