# Supplementary material for: Estimation of Newborn Risk for Child or Adolescent Obesity: Lessons from Longitudinal Birth Cohorts
Source: PLoS One. 2012 Nov 28;7(11):e49919. doi: 10.1371/journal.pone.0049919 (PMC3509134; doi:10.1371/journal.pone.0049919)
Supplement: Table S2 — Metabolic differences between overweight/obese adolescents with or without a history of childhood overweight/obesity in the NFBC1986. (DOC) [file pone.0049919.s003.doc]

|  | **Child overweight/obesity +** | **Child**  **overweight/obesity -** | P |
| --- | --- | --- | --- |
| Number | 331 | 347 |  |
| BMI | 28.62(3.96) | 26.54(2.44) | < 0.001 |
| Waist circumference | 91.17(10.60) | 83.92(8.13) | < 0.001 |
| Fasting plasma glucose (mmol/l) | 5.19(0.46) | 5.28(0.60) | 0.072 |
| Fasting serum insulin (UI/l) | 15.85(11.54) | 14.44(10.41) | 0.10 |
| Triglycerides (mg/dl) | 99.45(54.48) | 90.85(51.46) | 0.025 |
| HDL-cholesterol (mg/dl) | 48.06(9.61) | 49.47(10.24) | 0.33 |
| Systolic blood pressure | 123.66(13.64) | 122.69(12.65) | 0.27 |
| Diastolic blood pressure | 72.64(8.15) | 71.34(8.66) | 0.19 |
| Metabolic Syndrome^a^ carriers [Number (percentage)] | 36(11) | 9(2.5) | < 0.001 |

“child overweight/obesity +”= overweight/obese adolescents with a history of childhood overweight/obesity (overweight/obesity at 7 years of age);

“child overweight/obesity-” = overweight/obese adolescents without a history of childhood overweight/obesity (overweight/obesity at 7 years of age).

**^a^** *Metabolic syndrome was diagnosed if at least two other metabolic risk factors among impaired fasting glucose (fasting plasma glucose ≥ 100 mg/dl), high triglycerides (> 143 mg for boys and > 126 mg/dl for girls), low HDL-cholesterol (< 30 mg/dl for boys and < 35 mg/dl for girls) and systolic and/or diastolic hypertension (≥ 130 and 85 mmHg respectively) were present besides obesity. Triglycerides and HDL-cholesterol cut-offs corresponded to the 95° percentiles proposed by the AAP according to:* Tamir I, Heiss G, Glueck CJ et Al. Lipid and lipoprotein distributions in white children aged 6-19 years: the Lipid Research Clinics Program Prevalence Study. J Chronic Dis, 1981; 34: 27-39.
