# Supplementary material for: Estimation of Newborn Risk for Child or Adolescent Obesity: Lessons from Longitudinal Birth Cohorts
Source: PLoS One. 2012 Nov 28;7(11):e49919. doi: 10.1371/journal.pone.0049919 (PMC3509134; doi:10.1371/journal.pone.0049919)
Supplement: Table S3 — SNPs selected for building the genotype score with the relative genotyping quality control parameters. (DOC) [file pone.0049919.s004.doc]

| **SNP** | **Nearby Gene** | **Ref** | **P for**  **H-W equilibrium test** | **Genotyping success rate** | **Genotyping**  **consensus rate** | **Quality control criteria fulfilled** |
| --- | --- | --- | --- | --- | --- | --- |
| rs6496640 | *FTO* | 16 | 0.23 | 99.9 | 100 | Yes |
| rs6234 | *PCSK1* | 12 | 0.09 | 99.2 | 99.2 | Yes |
| rs6232 | *PCSK1* | 12 | 0.18 | 99.1 | 100 | Yes |
| rs7647305 | *ETV5* | 16,20 | 0.10 | 98.5 | 100 | Yes |
| rs4712652 | *PRL* | 17 | 0.12 | 99.4 | 100 | Yes |
| rs17498665 | *SH2B1* | 15,16,20 | 0.07 | 98.7 | 100 | Yes |
| rs10838738 | *MTCH2* | 15,20 | 0.31 | 99.2 | 99.6 | Yes |
| rs17782313 | *MC4R* | 14,20 | 0.34 | 99.5 | 100 | Yes |
| rs10913469 | *SEC16B* | 16,20 | 0.15 | 98.1 | 99.6 | Yes |
| rs10508503 | *PTER* | 17 | 0.42 | 98.8 | 100 | Yes |
| rs2815752 | *NEGR1* | 15,16,20 | 0.16 | 99.2 | 100 | Yes |
| rs7138803 | *FAIM2* | 16,20 | 0.11 | 99.8 | 100 | Yes |
| rs1421085 | *FTO* | 13,20 | 0.08 | 99.4 | 100 | Yes |
| rs6265 | *BDNF* | 16,20 | 0.12 | 99.2 | 100 | Yes |
| rs6013029 | *CTNNBL1* | 18 | 0.22 | 99.8 | 100 | Yes |
| rs2844479 | *AIF1* | 16 | 0.35 | 100 | 100 | Yes |
| rs1424233 | *MAF* | 17 | 0.41 | 99.7 | 100 | Yes |
| rs10938397 | *GNPDA2* | 15,20 | 0.38 | 99.6 | 99.6 | Yes |
| rs6548238 | *TMEM18* | 15,16,20 | 0.28 | 100 | 100 | Yes |
| rs925946 | *BDNF* | 16,20 | 0.10 | 99.2 | 100 | Yes |
| rs12145833 | *SDCCAG8* | 19 | 0.12 | 98.9 | 99.2 | Yes |
| rs1805081 | *NPC1* | 17 | 0.23 | 98.8 | 99.6 | Yes |
| rs11084753 | *KCDT15* | 15,16,20 | 0.14 | 99.1 | 100 | Yes |
| rs17150703 | *TNKS* | 19 | 0.20 | 99.8 | 100 | Yes |
| rs2890652 | *LRP1B* | 20 | 0.30 | 99.4 | 99.6 | Yes |
| rs4929949 | *RLP27A* | 20 | 0.24 | 99.6 | 100 | Yes |
| rs2112347 | *FLJ35779* | 20 | 0.18 | 99.9 | 100 | Yes |
| rs1514175 | *TNNI3K* | 20 | 0.43 | 99.9 | 100 | Yes |
| rs2183825 | *LRRN6C* | 20 | 0.33 | 100 | 99.2 | Yes |
| rs2241423 | *MAP2K5* | 20 | 0.32 | 100 | 99.2 | Yes |
| rs887912 | *FANCL* | 20 | 0.19 | 100 | 100 | Yes |
| rs12444979 | *GPRC5B* | 20 | 0.26 | 100 | 99.6 | Yes |
| rs4771122 | *MTIF3* | 20 | 0.09 | 99.9 | 99.6 | Yes |
| rs1555543 | *PTBP2* | 20 | 0.14 | 99.8 | 99.6 | Yes |
| rs6864049 | *ZNF608* | 20 | 0.16 | 99.8 | 99.6 | Yes |
| rs206936 | *NUDT3* | 20 | 0.21 | 99.9 | 100 | Yes |
| rs7640855 | *CADM2* | 20 | 0.17 | 99.9 | 99.2 | Yes |
| rs10134820 | *PRKD1* | 20 | 0.09 | 100 | 99.6 | Yes |
| rs13107325 | *SLC39A8* | 20 | 0.10 | 95.1 | 100 | Yes |
| rs713586 | *RBJ/ADCY3/POMC* | 20 | < 0.01 | 82 | 98.0 | No |
| rs3810291 | *TMEM160/ZC3H4* | 20 | < 0.001 | 72 | 97.0 | No |
| rs2287019 | *QPCTL/GIPR* | 20 | < 0.001 | 20 | - | No |
| rs987237 | *TFAP2B* | 20 | _ | 0 | - | No |
| rs10150332 | *NRXN3* | 20 | _ | 0 | - | No |
