# Supplementary material for: Estimation of Newborn Risk for Child or Adolescent Obesity: Lessons from Longitudinal Birth Cohorts
Source: PLoS One. 2012 Nov 28;7(11):e49919. doi: 10.1371/journal.pone.0049919 (PMC3509134; doi:10.1371/journal.pone.0049919)
Supplement: Table S4 — Associations between single SNPs and childhood obesity and overweight/obesity in the NFBC1986. (DOC) [file pone.0049919.s005.doc]

| **SNP** | **Nearby Gene** | **Ref** | **Effect allele** | **Other allele** | **OR for Obesity** | **95% C.I.** | **P** | **Statistical Power %** | **OR for OV/OB** | **95% C.I.** | **P** | **Statistical Power %** |
| --- | --- | --- | --- | --- | --- | --- | --- | --- | --- | --- | --- | --- |
| rs6496640 | *FTO* | 16 | A | G | 1.10 | 0.83-1.44 | 0.48 | 20 | 1.14 | 1.00-1.30 | 0.04 | NC |
| rs6234 | *PCSK1* | 12 | G | A | 1.23 | 0.93-1.63 | 0.14 | 22 | 1.09 | 0.94-1.25 | 0.22 | 24 |
| rs6232 | *PCSK1* | 12 | G | C | 0.83 | 0.40-1.70 | 0.61 | 8 | 0.88 | 0.62-1.25 | 0.49 | 17 |
| rs7647305 | *ETV5* | 16,20 | C | T | 1.24 | 0.87-1.77 | 0.22 | 5 | 1.12 | 0.95-1.32 | 0.16 | 6 |
| rs4712652 | *PRL* | 17 | A | G | 1.19 | 0.90-1.58 | 0.21 | 25 | 1.04 | 0.92-1.19 | 0.47 | NC |
| rs7498665 | *SH2B1* | 15,16,20 | G | A | 1.23 | 0.95-1.59 | 0.10 | 6 | 1.12 | 0.99-1.26 | 0.06 | 20 |
| rs10838738 | *MTCH2* | 15,20 | G | A | 1.17 | 0.91-1.52 | 0.21 | 5 | 1.06 | 0.94-1.20 | 0.32 | 7 |
| rs17782313 | *MC4R* | 14,20 | C | T | 1.35 | 1.01-1.84 | 0.04 | 12 | 1.27 | 1.09-1.48 | 0.002 | 12 |
| rs10913469 | *SEC16B* | 16,20 | C | T | 1.17 | 0.86-1.60 | 0.29 | 5 | 1.29 | 1.11-1.49 | 0.001 | 7 |
| rs10508503 | *PTER* | 17 | C | T | 0.66 | 0.45-1.00 | 0.05 | 29 | 0.87 | 0.70-1.07 | 0.18 | NC |
| rs2815752 | *NEGR1* | 15,16,20 | A | G | 1.04 | 0.79-1.36 | 0.75 | 6 | 1.23 | 1.08-1.41 | 0.001 | 12 |
| rs7138803 | *FAIM2* | 16,20 | A | G | 0.96 | 0.74-1.25 | 0.79 | 8 | 1.03 | 0.90-1.17 | 0.62 | 10 |
| rs1421085 | *FTO* | 13,20 | C | T | 1.29 | 0.99-1.69 | 0.06 | 49 | 1.19 | 1.04-1.35 | 0.007 | 82 |
| rs6265 | *BDNF* | 16,20 | G | A | 0.91 | 0.64-1.29 | 0.60 | 10 | 0.98 | 0.83-1.16 | 0.87 | NC |
| rs6013029 | *CTNNBL1* | 18 | T | G | 0.77 | 0.41-1.45 | 0.43 | 50 | 0.94 | 0.71-1.24 | 0.69 | NC |
| rs2844479 | *AIF1* | 16 | T | G | 1.01 | 0.76-1.33 | 0.94 | 7 | 1.02 | 0.89-1.16 | 0.78 | NC |
| rs1424233 | *MAF* | 17 | A | G | 1.09 | 0.84-1.41 | 0.49 | 68 | 1.06 | 0.94-1.10 | 0.31 | NC |
| rs10938397 | *GNPDA2* | 15,20 | G | A | 1.27 | 0.98-1.63 | 0.06 | 8 | 1.12 | 0.99-1.27 | 0.05 | 16 |
| rs6548238 | *TMEM18* | 15,16,20 | C | T | 1.27 | 0.86-1.87 | 0.21 | 17 | 1.13 | 0.95-1.35 | 0.14 | 32 |
| rs925946 | *BDNF* | 16,20 | T | G | 1.01 | 0.75-1.35 | 0.94 | 8 | 0.99 | 0.86-1.14 | 0.93 | 61 |
| rs12145833 | *SDCCAG8* | 19 | T | G | 1.14 | 0.77-1.69 | 0.50 | 15 | 0.84 | 0.71-1.00 | 0.06 | NC |
| rs1805081 | *NPC1* | 17 | A | G | 0.98 | 0.76-1.27 | 0.90 | 72 | 1.09 | 0.96-1.23 | 0.15 | NC |
| rs11084753 | *KCDT15* | 15,16,20 | G | A | 1.18 | 0.89-1.55 | 0.24 | 6 | 0.94 | 0.83-1.07 | 0.41 | 7 |
| rs17150703 | *TNKS* | 19 | A | G | 1.07 | 0.77-1.49 | 0.67 | 15 | 0.97 | 0.83-1.15 | 0.79 | NC |
| rs2890652 | *LRP1B* | 20 | C | T | 1.03 | 0.78-1.35 | 0.83 | 6 | 0.93 | 0.81-1.06 | 0.31 | 7 |
| rs4929949 | *RLP27A* | 20 | C | T | 0.87 | 0.67-1.12 | 0.28 | 5 | 1.03 | 0.91-1.17 | 0.54 | 5 |
| rs2112347 | *FLJ35779* | 20 | T | G | 0.89 | 0.68-1.15 | 0.38 | 7 | 0.99 | 0.87-1.12 | 0.87 | 10 |
| rs1514175 | *TNNI3K* | 20 | A | G | 1.16 | 0.90-1.50 | 0.23 | 6 | 1.17 | 1.03-1.32 | 0.01 | 10 |
| rs2183825 | *LRRN6C* | 20 | C | T | 0.97 | 0.74-1.26 | 0.84 | 5 | 1.05 | 0.93-1.20 | 0.37 | 5 |
| rs2241423 | *MAP2K5* | 20 | G | A | 1.04 | 0.72-1.49 | 0.81 | 6 | 1.00 | 0.84-1.19 | 0.96 | 7 |
| rs887912 | *FANCL* | 20 | T | C | 1.42 | 1.08-1.87 | 0.01 | 5 | 1.10 | 0.96-1.26 | 0.16 | 5 |
| rs12444979 | *GPRC5B* | 20 | C | T | 1.28 | 0.84-1.95 | 0.24 | 7 | 1.14 | 0.94-1.38 | 0.16 | 15 |
| rs4771122 | *MTIF3* | 20 | G | A | 1.02 | 0.78-1.34 | 0.84 | 6 | 1.00 | 0.87-1.13 | 0.99 | 7 |
| rs1555543 | *PTBP2* | 20 | C | A | 1.35 | 1.03-1.78 | 0.03 | 5 | 1.18 | 1.04-1.34 | 0.01 | 6 |
| rs6864049 | *ZNF608* | 20 | G | A | 0.83 | 0.64-1.08 | 0.17 | 6 | 1.04 | 0.91-1.17 | 0.53 | 8 |
| rs206936 | *NUDT3* | 20 | G | A | 0.99 | 0.74-1.34 | 0.98 | 5 | 1.14 | 0.99-1.31 | 0.07 | 7 |
| rs7640855 | *CADM2* | 20 | G | A | 1.05 | 0.73-1.50 | 0.77 | 5 | 0.99 | 0.84-1.17 | 0.92 | 6 |
| rs10134820 | *PRKD1* | 20 | T | C | 0.89 | 0.27-2.90 | 0.85 | 5 | 1.14 | 0.69-1.89 | 0.59 | 6 |
| rs13107325 | *SLC39A8* | 20 | T | C | 0.31 | 0.04-2.35 | 0.26 | 6 | 0.63 | 0.32-1.23 | 0.18 | 7 |

*OV/OB = overweight/obesity. Power estimates were performed according to published ORs, assuming an alpha error probability = 0.05.*

*Ref = bibliographic references related to SNPs discovery or SNPs/loci replication. NC = not computed because of the lack of any published OR for overweight/obesity. For SNPs associated with more than one reference, ORs issued from reference 20 were used for power calculations.*
