# Supplementary material for: Estimation of Newborn Risk for Child or Adolescent Obesity: Lessons from Longitudinal Birth Cohorts
Source: PLoS One. 2012 Nov 28;7(11):e49919. doi: 10.1371/journal.pone.0049919 (PMC3509134; doi:10.1371/journal.pone.0049919)
Supplement: Table S5 — Associations between single SNPs and adolescent obesity and overweight/obesity in the NFBC1986. (DOC) [file pone.0049919.s006.doc]

| **SNP** | **Nearby Gene** | **Ref** | **Effect allele** | **Other allele** | **OR for Obesity** | **95% C.I.** | **P** | **Statistical Power %** | **OR for OV/OB** | **95% C.I.** | **P** | **Statistical Power %** |
| --- | --- | --- | --- | --- | --- | --- | --- | --- | --- | --- | --- | --- |
| rs6496640 | *FTO* | 16 | A | G | 0.94 | 0.75-1.17 | 0.74 | 26 | 1.03 | 0.92-1.16 | 0.03 | NC |
| rs6234 | *PCSK1* | 12 | G | A | 0.93 | 0.73-1.19 | 0.97 | 28 | 1.05 | 0.92-1.19 | 0.26 | 28 |
| rs6232 | *PCSK1* | 12 | G | C | 1.47 | 0.88-2.46 | 0.15 | 11 | 1.16 | 0.86-1.55 | 0.25 | 20 |
| rs7647305 | *ETV5* | 16,20 | C | T | 1.13 | 0.85-1.50 | 0.39 | 10 | 1.00 | 0.86-1.15 | 0.90 | 8 |
| rs4712652 | *PRL* | 17 | A | G | 1.20 | 0.95-1.52 | 0.13 | 30 | 0.99 | 0.88-1.11 | 0.86 | NC |
| rs7498665 | *SH2B1* | 15,16,20 | G | A | 1.16 | 0.94-1.42 | 0.18 | 8 | 1.01 | 0.91-1.13 | 0.71 | 31 |
| rs10838738 | *MTCH2* | 15,20 | G | A | 1.20 | 0.97-1.48 | 0.08 | 7 | 1.01 | 0.90-1.13 | 0.67 | 10 |
| rs17782313 | *MC4R* | 14,20 | C | T | 1.34 | 1.04-1.73 | 0.02 | 17 | 1.33 | 1.16-1.53 | < 0.001 | 15 |
| rs10913469 | *SEC16B* | 16,20 | C | T | 1.35 | 1.06-1.73 | 0.01 | 8 | 1.33 | 1.17-1.52 | < 0.001 | 25 |
| rs10508503 | *PTER* | 17 | C | T | 0.80 | 0.57-1.12 | 0.23 | 39 | 0.92 | 0.76-1.11 | 0.50 | NC |
| rs2815752 | *NEGR1* | 15,16,20 | A | G | 1.17 | 0.94-1.46 | 0.14 | 9 | 1.16 | 1.03-1.31 | 0.009 | 12 |
| rs7138803 | *FAIM2* | 16,20 | A | G | 1.07 | 0.86-1.32 | 0.46 | 11 | 1.06 | 0.94-1.18 | 0.19 | 12 |
| rs1421085 | *FTO* | 13,20 | C | T | 1.39 | 1.11-1.73 | 0.01 | 52 | 1.30 | 1.16-1.46 | < 0.001 | 73 |
| rs6265 | *BDNF* | 16,20 | G | A | 0.92 | 0.68-1.23 | 0.72 | 14 | 0.93 | 0.80-1.09 | 0.32 | NC |
| rs6013029 | *CTNNBL1* | 18 | T | G | 0.60 | 0.33-1.06 | 0.10 | 42 | 0.68 | 0.51-1.00 | 0.05 | NC |
| rs2844479 | *AIF1* | 16 | T | G | 0.85 | 0.67-1.08 | 0.18 | 10 | 1.02 | 0.91-1.16 | 0.61 | NC |
| rs1424233 | *MAF* | 17 | A | G | 1.38 | 1.12-1.70 | 0.002 | 69 | 1.16 | 1.04-1.30 | 0.005 | NC |
| rs10938397 | *GNPDA2* | 15,20 | G | A | 1.22 | 0.99-1.50 | 0.10 | 12 | 1.08 | 0.97-1.21 | 0.18 | 18 |
| rs6548238 | *TMEM18* | 15,16,20 | C | T | 0.86 | 0.65-1.14 | 0.47 | 22 | 1.05 | 0.90-1.23 | 0.29 | 31 |
| rs925946 | *BDNF* | 16,20 | T | G | 1.21 | 0.96-1.52 | 0.16 | 23 | 1.01 | 0.89-1.14 | 0.99 | 54 |
| rs12145833 | *SDCCAG8* | 19 | T | G | 0.98 | 0.73-1.33 | 0.91 | 20 | 0.96 | 0.82-1.13 | 0.70 | NC |
| rs1805081 | *NPC1* | 17 | A | G | 0.97 | 0.79-1.19 | 0.87 | 73 | 1.04 | 0.93-1.17 | 0.34 | NC |
| rs11084753 | *KCDT15* | 15,16,20 | G | A | 0.90 | 0.73-1.12 | 0.42 | 7 | 1.05 | 0.94-1.18 | 0.33 | 9 |
| rs17150703 | *TNKS* | 19 | A | G | 0.88 | 0.66-1.18 | 0.37 | 20 | 0.97 | 0.83-1.12 | 0.53 | NC |
| rs2890652 | *LRP1B* | 20 | C | T | 0.78 | 0.61-1.00 | 0.05 | 6 | 0.87 | 0.77-0.99 | 0.06 | 8 |
| rs4929949 | *RLP27A* | 20 | C | T | 0.91 | 0.74-1.12 | 0.50 | 7 | 1.01 | 0.91-1.13 | 0.74 | 6 |
| rs2112347 | *FLJ35779* | 20 | T | G | 1.07 | 0.87-1.33 | 0.60 | 9 | 0.98 | 0.87-1.10 | 0.53 | 12 |
| rs1514175 | *TNNI3K* | 20 | A | G | 1.19 | 0.97-1.46 | 0.15 | 8 | 1.14 | 1.02-1.27 | 0.04 | 12 |
| rs2183825 | *LRRN6C* | 20 | C | T | 1.02 | 0.82-1.26 | 0.90 | 8 | 1.06 | 0.94-1.19 | 0.34 | 11 |
| rs2241423 | *MAP2K5* | 20 | G | A | 1.13 | 0.83-1.52 | 0.47 | 9 | 1.02 | 0.87-1.19 | 0.86 | 10 |
| rs887912 | *FANCL* | 20 | T | C | 1.05 | 0.83-1.32 | 0.64 | 7 | 1.04 | 0.92-1.18 | 0.38 | 9 |
| rs12444979 | *GPRC5B* | 20 | C | T | 1.20 | 0.86-1.69 | 0.24 | 9 | 1.19 | 1.00-1.42 | 0.07 | 16 |
| rs4771122 | *MTIF3* | 20 | G | A | 1.02 | 0.82-1.27 | 0.71 | 8 | 1.04 | 0.93-1.17 | 0.35 | 9 |
| rs1555543 | *PTBP2* | 20 | C | A | 1.05 | 0.85-1.30 | 0.54 | 25 | 1.21 | 1.08-1.36 | 0.001 | 10 |
| rs6864049 | *ZNF608* | 20 | G | A | 1.01 | 0.82-1.24 | 0.87 | 7 | 1.00 | 0.89-1.12 | 0.99 | 10 |
| rs206936 | *NUDT3* | 20 | G | A | 1.05 | 0.82-1.34 | 0.69 | 7 | 1.05 | 0.92-1.20 | 0.40 | 8 |
| rs7640855 | *CADM2* | 20 | G | A | 0.83 | 0.64-1.10 | 0.23 | 6 | 0.82 | 0.71-0.95 | 0.01 | 8 |
| rs10134820 | *PRKD1* | 20 | T | C | 0.42 | 0.10-1.76 | 0.17 | 6 | 0.74 | 0.43-1.28 | 0.28 | 7 |
| rs13107325 | *SLC39A8* | 20 | T | C | 1.06 | 0.43-2.62 | 0.81 | 10 | 0.61 | 0.33-1.12 | 0.08 | 8 |

*OV/OB = overweight/obesity. Power estimates were performed according to published ORs, assuming an alpha error probability = 0.05.*

*Ref = bibliographic references related to SNPs discovery or SNPs/loci replication. NC = not computed because of the lack of any published OR for overweight/obesity. For SNPs associated with more than one reference, ORs issued from reference 20 were used for power calculations.*
