# Supplementary material for: Estimation of Newborn Risk for Child or Adolescent Obesity: Lessons from Longitudinal Birth Cohorts
Source: PLoS One. 2012 Nov 28;7(11):e49919. doi: 10.1371/journal.pone.0049919 (PMC3509134; doi:10.1371/journal.pone.0049919)
Supplement: Table S6 — Associations between single SNPs and persistent obesity and overweight/obesity in the NFBC1986. (DOC) [file pone.0049919.s007.doc]

| **SNP** | **Nearby Gene** | **Ref** | **Effect allele** | **Other allele** | **OR for Obesity** | **95% C.I.** | **P** | **Statistical Power %** | **OR for OV/OB** | **95% C.I.** | **P** | **Statistical Power %** |
| --- | --- | --- | --- | --- | --- | --- | --- | --- | --- | --- | --- | --- |
| rs6496640 | *FTO* | 16 | A | G | 1.25 | 0.80-1.97 | 0.31 | 10 | 1.20 | 1.00-1.43 | 0.05 | NC |
| rs6234 | *PCSK1* | 12 | G | A | 0.86 | 0.52-1.41 | 0.56 | 11 | 1.04 | 0.86-1.25 | 0.64 | 17 |
| rs6232 | *PCSK1* | 12 | G | C | 1.33 | 0.44-4.03 | 0.61 | 6 | 1.11 | 0.72-1.73 | 0.62 | 11 |
| rs7647305 | *ETV5* | 16,20 | C | T | 1.49 | 0.79-2.81 | 0.21 | 4 | 1.01 | 0.81-1.25 | 0.89 | 9 |
| rs4712652 | *PRL* | 17 | A | G | 1.09 | 0.69-1.72 | 0.69 | 12 | 1.01 | 0.85-1.21 | 0.85 | NC |
| rs7498665 | *SH2B1* | 15,16,20 | G | A | 1.10 | 0.72-1.68 | 0.65 | 7 | 1.10 | 0.93-1.29 | 0.24 | 8 |
| rs10838738 | *MTCH2* | 15,20 | G | A | 1.31 | 0.86-2.00 | 0.20 | 5 | 1.04 | 0.88-1.22 | 0.64 | 6 |
| rs17782313 | *MC4R* | 14,20 | C | T | 1.60 | 0.98-2.60 | 0.04 | 8 | 1.41 | 1.15-1.72 | 0.001 | 9 |
| rs10913469 | *SEC16B* | 16,20 | C | T | 1.59 | 1.00-2.53 | 0.05 | 5 | 1.40 | 1.16-1.69 | < 0.001 | 6 |
| rs10508503 | *PTER* | 17 | C | T | 0.60 | 0.33-1.08 | 0.09 | 14 | 0.84 | 0.64-1.11 | 0.23 | NC |
| rs2815752 | *NEGR1* | 15,16,20 | A | G | 1.53 | 0.95-2.46 | 0.07 | 5 | 1.31 | 1.09-1.56 | 0.003 | 9 |
| rs7138803 | *FAIM2* | 16,20 | A | G | 1.00 | 0.65-1.55 | 0.97 | 6 | 1.02 | 0.86-1.20 | 0.80 | 8 |
| rs1421085 | *FTO* | 13,20 | C | T | 1.10 | 0.70-1.72 | 0.65 | 22 | 1.36 | 1.15-1.62 | < 0.001 | 57 |
| rs6265 | *BDNF* | 16,20 | G | A | 0.95 | 0.52-1.74 | 0.88 | 7 | 0.91 | 0.72-1.14 | 0.42 | NC |
| rs6013029 | *CTNNBL1* | 18 | T | G | 0.45 | 0.11-1.84 | 0.27 | 23 | 0.60 | 0.39-0.95 | 0.029 | NC |
| rs2844479 | *AIF1* | 16 | T | G | 0.91 | 0.57-1.44 | 0.69 | 6 | 0.96 | 0.80-1.15 | 0.66 | NC |
| rs1424233 | *MAF* | 17 | A | G | 1.16 | 0.76-1.78 | 0.47 | 32 | 1.06 | 0.90-1.25 | 0.47 | NC |
| rs10938397 | *GNPDA2* | 15,20 | G | A | 1.17 | 0.77-1.78 | 0.45 | 6 | 1.14 | 0.97-1.35 | 0.09 | 12 |
| rs6548238 | *TMEM18* | 15,16,20 | C | T | 0.78 | 0.45-1.35 | 0.37 | 9 | 1.05 | 0.83-1.32 | 0.65 | 20 |
| rs925946 | *BDNF* | 16,20 | T | G | 1.16 | 0.73-1.84 | 0.52 | 6 | 1.02 | 0.85-1.23 | 0.79 | 38 |
| rs12145833 | *SDCCAG8* | 19 | T | G | 0.98 | 0.53-1.81 | 0.97 | 9 | 0.86 | 0.68-1.08 | 0.20 | NC |
| rs1805081 | *NPC1* | 17 | A | G | 0.77 | 0.51-1.18 | 0.24 | 34 | 1.01 | 0.86-1.19 | 0.86 | NC |
| rs11084753 | *KCDT15* | 15,16,20 | G | A | 1.09 | 0.70-1.72 | 0.68 | 5 | 1.06 | 0.89-1.27 | 0.46 | 6 |
| rs17150703 | *TNKS* | 19 | A | G | 0.82 | 0.45-1.49 | 0.52 | 9 | 0.95 | 0.76-1.18 | 0.65 | NC |
| rs2890652 | *LRP1B* | 20 | C | T | 1.05 | 0.67-1.64 | 0.82 | 5 | 0.88 | 0.73-1.05 | 0.17 | 6 |
| rs4929949 | *RLP27A* | 20 | C | T | 0.78 | 0.51-1.19 | 0.26 | 5 | 1.02 | 0.86-1.20 | 0.79 | 5 |
| rs2112347 | *FLJ35779* | 20 | T | G | 0.82 | 0.53-1.25 | 0.35 | 6 | 0.92 | 0.78-1.09 | 0.37 | 8 |
| rs1514175 | *TNNI3K* | 20 | A | G | 1.33 | 0.87-2.03 | 0.18 | 5 | 1.12 | 0.95-1.32 | 0.16 | 8 |
| rs2183825 | *LRRN6C* | 20 | C | T | 0.87 | 0.55-1.35 | 0.53 | 5 | 0.96 | 0.81-1.14 | 0.68 | 5 |
| rs2241423 | *MAP2K5* | 20 | G | A | 1.73 | 0.85-3.50 | 0.12 | 5 | 1.01 | 0.80-1.27 | 0.89 | 6 |
| rs887912 | *FANCL* | 20 | T | C | 1.34 | 0.86-2.11 | 0.19 | 5 | 1.21 | 1.01-1.45 | 0.03 | 5 |
| rs12444979 | *GPRC5B* | 20 | C | T | 0.92 | 0.49-1.73 | 0.80 | 6 | 1.27 | 0.97-1.66 | 0.07 | 10 |
| rs4771122 | *MTIF3* | 20 | G | A | 1.01 | 0.64-1.58 | 0.95 | 5 | 1.01 | 0.85-1.20 | 0.85 | 6 |
| rs1555543 | *PTBP2* | 20 | C | A | 1.24 | 0.79-1.93 | 0.33 | 5 | 1.34 | 1.12-1.59 | 0.001 | 5 |
| rs6864049 | *ZNF608* | 20 | G | A | 0.76 | 0.49-1.17 | 0.22 | 5 | 0.98 | 0.83-1.16 | 0.89 | 6 |
| rs206936 | *NUDT3* | 20 | G | A | 0.95 | 0.57-1.58 | 0.85 | 5 | 1.13 | 0.94-1.37 | 0.18 | 6 |
| rs7640855 | *CADM2* | 20 | G | A | 1.23 | 0.66-2.30 | 0.50 | 5 | 0.95 | 0.76-1.19 | 0.70 | 6 |
| rs10134820 | *PRKD1* | 20 | T | C | 0.00 | NC | 0.99 | 5 | 0.63 | 0.27-1.46 | 0.28 | 5 |
| rs13107325 | *SLC39A8* | 20 | T | C | 0.04 | 0.00-528 | 0.51 | 5 | 0.50 | 0.18-1.40 | 0.19 | 6 |

*OV/OB = overweight/obesity. Power estimates were performed according to published ORs, assuming an alpha error probability = 0.05.*

*Ref = bibliographic references related to SNPs discovery or SNPs/loci replication. NC = not computed because of the lack of any published OR for overweight/obesity. For SNPs associated with more than one reference, ORs issued from reference 20 were used for power calculations.*
