# Supplementary material for: Estimation of Newborn Risk for Child or Adolescent Obesity: Lessons from Longitudinal Birth Cohorts
Source: PLoS One. 2012 Nov 28;7(11):e49919. doi: 10.1371/journal.pone.0049919 (PMC3509134; doi:10.1371/journal.pone.0049919)
Supplement: Table S7 — Association, discrimination and calibration parameters of the 39-SNPs genetic score predicting the six obesity outcomes in the NFBC1986. (DOC) [file pone.0049919.s008.doc]

|  | **OR (95% C.I.)** | **AUC (95% C.I.)** | **Calibration Chi Square^a^** |
| --- | --- | --- | --- |
| **Childhood**  **Obesity** | 1.09 (1.04 – 1.14)  P = 0.001 | 0.59 (0.54 – 0.64)  P < 0.001 | 6.74  P = 0.56 |
| **Childhood**  **Overweight/obesity** | 1.05 (1.03 – 1.08)  P < 0.001 | 0.57 (0.55 – 0.59)  P < 0.001 | 6.17  P = 0.62 |
| **Adolescent**  **Obesity** | 1.07 (1.03 – 1.11)  P < 0.001 | 0.58 (0.54 – 0.62)  P < 0.001 | 3.76  P = 0.87 |
| **Adolescent**  **Overweight/obesity** | 1.05 (1.03 – 1.08)  P < 0.001 | 0.56 (0.54 – 0.58)  P < 0.001 | 8.31  P = 0.40 |
| **Persistent Childhood**  **Obesity** | 1.08 (1.00 – 1.16)  P = 0.04 | 0.58 (0.50 – 0.66)  P < 0.001 | 2.64  P = 0.95 |
| **Persistent Childhood**  **Overweight/obesity** | 1.08 (1.05 – 1.11)  P < 0.001 | 0.58 (0.55 – 0.62)  P < 0.001 | 9.73  P = 0.28 |

**^a^ *The calibration chi square corresponds to the Hosmer-Lemeshow test, that compares the expected to the observed event rate through ten classes (deciles) of estimated risk. The DF (degree of freedom) of the test is equal to K-2 = 8 (K = N of classes, 2 = N of unknown constants, i.e. sample size and event proportion).***
