# Supplementary material for: Estimation of Newborn Risk for Child or Adolescent Obesity: Lessons from Longitudinal Birth Cohorts
Source: PLoS One. 2012 Nov 28;7(11):e49919. doi: 10.1371/journal.pone.0049919 (PMC3509134; doi:10.1371/journal.pone.0049919)
Supplement: Table S8 — Association, discrimination and calibration parameters of the genetic score composed of the 20 “childhood obesity SNPs”a predicting the six obesity outcomes in the NFBC1986. (DOC) [file pone.0049919.s009.doc]

|  | **OR (95% C.I.)** | **AUC (95% C.I.)** | **Calibration Chi Square^b^** |
| --- | --- | --- | --- |
| **Childhood**  **Obesity** | 1.12 (1.09 – 1.15)  P = 0.001 | 0.59 (0.54 – 0.64)  P < 0.001 | 44.69  P < 0.001 |
| **Childhood**  **Overweight/obesity** | 1.08 (1.05 – 1.11)  P < 0.001 | 0.57 (0.55 – 0.59)  P < 0.001 | 9.17  P = 0.24 |
| **Adolescent**  **Obesity** | 1.11 (1.08 – 1.13)  P < 0.001 | 0.57 (0.53 – 0.61)  P < 0.001 | 10.54  P = 0.16 |
| **Adolescent**  **Overweight/obesity** | 1.08 (1.05 – 1.11)  P < 0.001 | 0.57 (0.55 – 0.59)  P < 0.001 | 5.20  P = 0.63 |
| **Persistent Childhood**  **Obesity** | 1.13 (1.02 – 1.24)  P = 0.02 | 0.59 (0.50 – 0.68)  P = 0.028 | 8.04  P = 0.33 |
| **Persistent Childhood**  **Overweight/obesity** | 1.11 (1.07 – 1.15)  P < 0.001 | 0.59 (0.56 – 0.62)  P < 0.001 | 3.68  P = 0.81 |

**^a^ SNPs associated with childhood obesity:** rs6496640 (FTO), rs6234 (PCSK1), rs6232 (PCSK1), rs7647305 (ETV5), rs17498665 (SH2B1), rs10838738 (MTCH2), rs17782313 (MC4R), rs10913469 (SEC16B), rs10508503 (PTER), rs2815752 (NEGR1), rs7138803 (FAIM2), rs1421085 (FTO), rs6265 (BDNF), rs1424233 (MAF), rs10938397 (GNPDA2), rs6548238 (TMEM18), rs925946 (BDNF), rs12145833 (SDCCAG8), rs1805081 (NPC1), rs11084753 (*KCDT15*)*.*

**^b^ *The calibration chi square corresponds to the Hosmer-Lemeshow test, that compares the expected to the observed event rate through ten classes (deciles) of estimated risk. The DF (degree of freedom) of the test is equal to K-2 = 8 (K = N of classes, 2 = N of unknown constants, i.e. sample size and event proportion).***
