# Supplementary material for: Estimation of Newborn Risk for Child or Adolescent Obesity: Lessons from Longitudinal Birth Cohorts
Source: PLoS One. 2012 Nov 28;7(11):e49919. doi: 10.1371/journal.pone.0049919 (PMC3509134; doi:10.1371/journal.pone.0049919)
Supplement: Dataset S1 — Equations predicting the obesity phenotypes from traditional risk factors. (DOC) [file pone.0049919.s010.doc]

**Dataset S1:**

**Equations predicting the obesity phenotypes from traditional risk factors:**

***NFBC 1986:***

***Risk for childhood obesity*** = **e**(-10.61+0.12 x maternal BMI + 0.18 x paternal BMI -0.31 x number of household members - 0.69 x maternal occupation + 0.61 x gestational smoking* + 0.75 x birth weight) **/** (**1** + **e**(-10.61+0.12 x maternal BMI + 0.18 x paternal BMI -0.31 x number of household members - 0.69 x maternal occupation + 0.61 x gestational smoking* + 0.75 x birth weight))

***Risk for adolescence obesity*** = **e**(-7.64+0.17 x maternal BMI + 0.15 x paternal BMI -0.19 x number of household members - 0.75 x maternal occupation + 0.03 x gestational weight gain) **/** (**1** + **e**(-7.64+0.17 x maternal BMI + 0.15 x paternal BMI -0.19 x number of household members - 0.75 x maternal occupation + 0.03 x gestational weight gain))

***Risk for persistent childhood obesity*** = **e**(-12.66+0.13 x maternal BMI + 0.21 x paternal BMI -0.38 x number of household members - 1.17 x maternal occupation + 1.45 x single parenthood* + 0.83 x birth weight) **/** (**1** **e**(-12.66+0.13 x maternal BMI + 0.21 x paternal BMI -0.38 x number of household members - 1.17 x maternal occupation + 1.45 x single parenthood* + 0.83 x birth weight))

***Risk for childhood overweight/obesity*** = **e**(-9.19+0.12 x maternal BMI + 0.11 x paternal BMI -0.12 x number of household members + 0.25 x maternal smoking* + 0.02 x gestational weight gain + 0.36 x birth weight) **/** (**1** + **e**(-9.19+0.12 x maternal BMI + 0.11 x paternal BMI -0.12 x number of household members + 0.25 x maternal smoking* + 0.02 x gestational weight gain + 0.36 x birth weight))

***Risk for adolescence overweight/obesity*** = **e**(-8.21+ 0.16 x maternal BMI + 0.12 x paternal BMI -0.1 x number of household members - 0.27 x maternal occupation + 0.22 x maternal smoking* + 0.02 x gestational weight gain + 0.29 x birth weight) **/** (**1** + **e**(-8.21+0.16 x maternal BMI + 0.12 x paternal BMI -0.1 x number of household members - 0.27 x maternal occupation + 0.22 x maternal smoking* + 0.02 x gestational weight gain + 0.29 x birth weight))

***Risk for persistent childhood overweight/obesity*** = **e**(-9.58 + 0.17 x maternal BMI + 0.13 x paternal BMI - 0.56 x maternal occupation + 0.37 x gestational smoking* + 0.34 x birth weight) **/** (**1** + **e**(-9.58 + 0.17 x maternal BMI + 0.13 x paternal BMI - 0.56 x maternal occupation + 0.37 x gestational smoking* + 0.34 x birth weight))

***VENETO:***

***Risk for childhood obesity*** = **e**(-8.98+0.12 x maternal BMI + 0.13 x paternal BMI - 0.46 x female gender*) **/** (**1** + **e**(-8.98+0.12 x maternal BMI + 0.13 x paternal BMI - 0.46 x female gender*))

***VIVA:***

***Risk for childhood obesity*** = **e**(-8.12 + 0.10 x maternal BMI + 0.11 x paternal BMI + 0.90 x gestational smoking* + 0.05 x gestational weight gain + 0.60 x Black race* - 0.49 x White race* + 0.16 x Hispanic race* - 1.15 x Asian race*) **/ (1 + e**(-8.12 + 0.10 x maternal BMI + 0.11 x paternal BMI + 0.90 x gestational smoking* + 0.05 x gestational weight gain + 0.60 x Black race* - 0.49 x White race* + 0.16 x Hispanic race *- 1.15 x Asian race*))

*no=0, yes=1
